# Associations between null mutations in *GSTT1* and *GSTM1,* the *GSTP1* Ile^105^Val polymorphism, and mortality in breast cancer survivors

**DOI:** 10.1186/2193-1801-2-450

**Published:** 2013-09-11

**Authors:** Catherine Duggan, Rachel Ballard-Barbash, Richard N Baumgartner, Kathy B Baumgartner, Leslie Bernstein, Anne McTiernan

**Affiliations:** Public Health Sciences, Fred Hutchinson Cancer Research Center, Seattle, WA USA; Division of Cancer Control and Population Sciences, National Cancer Institute, Bethesda, MD USA; Department of Epidemiology and Population Health, School of Public Health and Information Sciences, University of Louisville, Louisville, KY USA; Division of Population Sciences, City of Hope National Medical Center, Duarte, CA USA

**Keywords:** Glutathione-S-transferases, GSTT1, GSTM1, GSTP1, Polymorphisms, Breast cancer survival, Mortality

## Abstract

**Purpose:**

Here we assessed associations between null mutations in glutathione-S-transferase (GST)*T1* and *GSTM1* genes, and the rs1695 polymorphism in *GSTP1* (Ile^105^Val), and risk of breast cancer-specific (n=45) and all-cause (n=99) mortality in a multiethnic, prospective cohort of 533 women diagnosed with stage I-IIIA breast cancer in 1995–1999, enrolled in the Health, Eating, Activity, and Lifestyle (HEAL) Study.

**Methods:**

We measured the presence of the null mutation in *GSTT1* and *GSTM1*, and the rs1695 polymorphism in *GSTP1* by polymerase chain reaction. We assessed associations between breast-cancer specific and all-cause mortality using Cox proportional hazards models.

**Results:**

Participants with ER-negative tumors were more likely to be *GSTT1* null (χ^2^=4.52; P=0.03), and African American women were more likely to be *GSTM1* null (χ^2^=34.36; P<0.0001). Neither *GSTM1* nor *GSTT1* null mutations were associated with breast cancer-specific or all-cause mortality. In a model adjusted for body mass index, race/ethnicity, tumor stage and treatment received at diagnosis, the variant Val allele of rs1695 was associated with increased risk of all-cause (HR=1.81, 95% CI 1.16-2.82, P=0.008), but not breast cancer-specific mortality. The GSTT1 null mutation was associated with significantly higher levels of C-reactive protein.

**Conclusions:**

GSTM1 and GSTT1 null genotypes had no effect on outcome; however the variant allele of rs1695 appears to confer increased risk for all-cause mortality in breast-cancer survivors.

Given the limited sample size of most studies examining associations between GST polymorphisms with breast cancer survival, and the lack of women undergoing more contemporary treatment protocols (treated prior to 1999), it may be helpful to re-examine this issue among larger samples of women diagnosed after the late 1990s, who all received some form of chemotherapy or radiotherapy.

## Introduction

The Glutathione-S Transferases (GST) are a phase II superfamily of cytosolic, mitochondrial and microsomal enzymes that catalyze the conjugation of reduced glutathione to electrophilic centers on a variety of substrates (Strange et al. 2001). This activity is acts a detoxification step for a variety of endogenous molecules and xenobiotics, including chemotherapeutic drugs. The mammalian cytosolic GSTs comprise 6 classes of dimeric isoenzymes alpha (α), mu (μ), pi (π), theta (τ), zeta (ζ) and omega (ω). GST-μ, GST-τ, and GST-π are encoded by the *GSTM1*, *GSTT1*, and *GSTP1* genes, respectively; and these 3 genes have been studied in association with genetic susceptibility to cancer (Strange & Fryer 1999; Spurdle et al. 2010).

Homozygous deletion of the *GSTM1* and *GSTT1* genes (null genotype), are associated with a lack of enzyme function and increased vulnerability to cytogenetic damage (Seidegard et al. 1988). Individuals who have deletions in *GSTM1* or *GSTT1* may therefore be at increased cancer risk (Strange & Fryer 1999; Rebbeck 1997).

The GST π (P1) polymorphism (rs1695; an A→G transition at position 313) results in an Ile→Val change at codon 105 (Ile^105^Val). The variant allele is associated with lower substrate-specific catalytic activity, including towards the alkylating anticancer agent chlorambucil (Hayes & Strange 2000; Pandya et al. 2000; Srivastava et al. 1999). A limited number of studies with conflicting results have investigated the association between polymorphisms in *GST* genes and mortality in breast cancer patients. The majority of these studied patients diagnosed prior to 1999. Five of six studies have samples of women undergoing chemotherapy and/or radiotherapy, and most examined only one *GST* gene (usually *GSTP1*). Four of the six (Ambrosone et al. 2001; Bewick et al. 2008; Lizard-Nacol et al. 1999; Sweeney et al. 2000) were based on small samples of patients (N<100; (Bewick et al. 2008; Lizard-Nacol et al. 1999) N=240-250 (Ambrosone et al. 2001; Sweeney et al. 2000)). One large study of 2430 breast cancer patients was comprised of women with early stage disease (94%) who were unlikely to have undergone chemotherapy, (Goode et al. 2002) and found no association with the only GST examined (*GSTP1*) and survival. One other large study of 1034 women from Shanghai, China, all treated with adjuvant chemotherapy, found a reduction in risk with the variant *GSTP1* Val allele but no association with either *GSTT1* or *GSTM1* and risk of death (Yang et al. 2005). In two reports based on the same sample, women with breast cancer with null mutations for *GSTM1* and *GSTT1* had reduced risk of death compared to women with alleles present, (Ambrosone et al. 2001) and a reduction in mortality risk for women homozygous for the variant *GSTP1* Val allele compared to those homozygous for the Ile allele (Sweeney et al. 2000; Yang et al. 2005). Finally, 2 other small studies examined associations between one GST polymorphism among women treated with high dose chemotherapy, one reported no association between survival and *GSTM1* null; (Lizard-Nacol et al. 1999) another, that the *GSTP1* Val/Val polymorphism was non-significantly associated with worse overall survival (Bewick et al. 2008).

Two studies (one in smokers, and the other in patients with diabetes), reported an association between *GSTT1* and *GSTM1* null mutations and lower levels of the inflammatory biomarker CRP, (Hayek et al. 2006; Miller et al. 2003) itself associated with poor survival (Pierce et al. 2009a). We thus examined this association in the Health, Eating, Activity and Lifestyle (HEAL) study.

We extend prior research by examining the association between three different GST isoenzymes (null mutations in *GSTM1* and *GSTT1*, the Ile^105^Val polymorphism in *GSTP1*), and all-cause and breast-cancer specific mortality in a multi-ethnic cohort of breast cancer survivors drawn from population-based cancer registries. This sample of breast cancer patients diagnosed from 1995–1999 includes a larger number of women undergoing chemotherapy and/or radiotherapy than most prior studies (Ambrosone et al. 2001; Bewick et al. 2008; Lizard-Nacol et al. 1999; Sweeney et al. 2000) and reflects more contemporary therapy regimens than those based on women treated in the mid-1980s-mid-1990s (Ambrosone et al. 2001; Bewick et al. 2008; Lizard-Nacol et al. 1999; Sweeney et al. 2000).

## Materials and methods

### Study setting, participants, and recruitment

The HEAL Study is a multicenter, multiethnic prospective cohort study which enrolled 1,183 women diagnosed with breast cancer, to evaluate effects of diet, weight, physical activity, lifestyle, hormones or other exposures on breast-cancer prognosis. Aims, study design and recruitment procedures have been published previously (McTiernan et al. 2003).

Briefly, women were recruited through Surveillance, Epidemiology, and End Results (SEER) registries in New Mexico (NM), Los Angeles County (CA), and western Washington (WA). Baseline surveys were conducted on average 6-months post-diagnosis. In NM, we recruited 615 women, ≥18 years, diagnosed with *in situ* to Stage IIIA breast cancer between 1996–1999. In WA, we recruited 202 women, aged 40–64 years, diagnosed with Stage 0-Stage IIIA breast cancer between 1997–1998. In CA, we recruited 366 Black women aged 35–64 years, with Stage 0-Stage IIIA breast cancer, who had participated in the Los Angeles portion of the Women’s Contraceptive and Reproductive Experiences Study, diagnosed with breast cancer between 1995–1998. Recruitment was restricted in WA and CA to women aged 35–64 at diagnosis because of competing studies and parent study design. The study was performed with the approval of the Institutional Review Boards of participating centers, in accordance with an assurance filed with and approved by the U.S. Department of Health and Human Services. Written informed consent was obtained from each subject.

944 women completed in-person interviews approximately 30-months following their first interview; 726 women were genotyped; we excluded 169 women with a diagnosis of Stage 0 (*in situ*) disease, and 24 women with non-fatal breast cancer events <9 months before their 24-month interview dates to avoid potential confounding from possible recent treatment. The final sample size is 533.

## Data collection and covariates

### Specimens

DNA was extracted from peripheral blood leukocytes, which was processed within 3 hours of collection, and stored at -80° C until analysis (Abrahamson et al. 2007).

### SNP analysis

*GSTT1*, *GSTP1* and *GSTM1* were genotyped at Albany Molecular Research in Bothell, Washington. The presence/absence of the *GSTM1* and *GSTT1* alleles were detected by PCR, and the Taqman allelic discrimination method (Applied Biosystems, Foster City, CA) was used to differentiate *GSTP1* genotypes (Kelada et al. 2003). We included 10% replica samples and genotype concordance was 100%. The *GSTM1* and *GSTT1* mutations were classified as *GST* null or *GST* positive genotypes.

### Covariates and inflammatory biomarkers

Standardized questionnaire information including medical history, demographic and lifestyle information, was collected at approximately 6- and 30-months post-diagnosis. With participants wearing light indoor clothing and no shoes, weight was measured to the nearest 0.1 kg, and height to the nearest 0.1 cm. All measurements were performed twice, and averaged. Body mass index (BMI) was calculated as kg/m^2^. A race/ethnicity/study site 4-category variable was created to adjust for race and site-associated confounding as these were highly correlated. The variable had 4 categories: Non-Hispanic whites (NM); non-Hispanic whites (WA); Hispanics; and African Americans.

Serum levels of C-reactive protein (CRP) were measured as described previously (Pierce et al. 2009b). CRP was non-normally distributed and was log-transformed.

### Stage of disease and cancer treatment

Participants were classified as having Stage 0 (*in situ*), Stage I (localized) or Stage II-IIIA (regional) breast cancer based on AJCC stage of disease classification contained within SEER. This analysis includes only women with Stage I-IIIa at diagnosis because few deaths occurred in women with Stage 0 disease. Estrogen receptor (ER) status was categorized as positive, negative, or unknown/borderline. Treatment and additional clinical data were obtained from medical record reviews. Treatment was categorized into 3 groups: surgery only, surgery plus radiation, or surgery with any chemotherapy with or without radiation.

### Outcome assessment

Information on vital status and cause of death codes were acquired from linkages with SEER databases. If alive, individuals were followed through their last follow-up assessment or SEER vital status update, whichever was most recent. All-cause mortality was defined as time from study enrollment to death from any cause, or end of follow-up (31 December 2009). Breast cancer-specific mortality was defined as death from breast cancer or end of follow-up, with the same intervals as for all-cause mortality.

## Statistical analysis

Differences in distribution of continuous variables between genotypes were estimated using analysis of variance (ANOVA). Differences in distributions of categorical variables were compared using the Chi-square test. As the numbers of patients homozygous for the *GSTP1* variant allele were few, heterozygous and homozygous variant allele groups were combined (recessive model). Hazard ratios (HR) and 95% confidence intervals (CI) for breast cancer-specific or all-cause mortality were based on the partial likelihood for Cox's proportional hazards model (Cox 1972). The proportional hazard assumption was tested using Schoenfeld residuals, and no violation of the proportionality assumption was found. Age was used as the underlying time variable, with entry and exit time defined as the participant’s age at the baseline interview, and age at death from either breast cancer or any cause, or end of follow-up, respectively.

We based variable inclusion on a likelihood ratio test, with the following covariates included in models: race/ethnicity/study-site (to adjust for different distributions of race/ethnicity by study site); BMI (categorical <18.5 kg/m^2^; ≥18.5 and <25 kg/m^2^; ≥25 and <40 kg/m^2^; ≥40 kg/m^2^); SEER summary tumor stage (local vs. regional) and treatment received at diagnosis (surgery; surgery+radiotherapy; chemotherapy). Covariates considered but not included in the final model (they did not significantly change the likelihood ratio score): menopausal status, education, smoking status, tamoxifen use, and ER status. The Wald statistic was used to test for trend across levels.

We determined whether the association of *GST* variants with outcome was the same across subgroup categories, using a test of homogeneity of trends across groups; specifically stage, ER status; and treatment received. Due to small numbers of events in premenopausal participants, we did not compare pre- and postmenopausal subgroups.

All p-values are two-sided. Analyses were performed using STATA 11 (Statacorp, TX USA).

## Results

Mean age of participants was 57.6 years (Table 1); more than half (52.5%) of participants carried at least one *GSTM1* null mutation, and significantly more African American women carried it compared to NHW or Hispanics (χ^2^=34.36 P<0.0001). 79.6% of participants carried at least one *GSTT1* null mutation; there was no difference in the proportion of carriers and non-carriers across racial/ethnic groups. For the *GSTP1* Ile^105^Val polymorphism, 58.2% of participants carried at least one variant allele; there were no differences across racial/ethnic groups. The *GSTP1*^105^Ile/Val polymorphism was in Hardy-Weinberg equilibrium (P>0.05). Median follow-up time was 11.29 years.

**Table 1:** **Characteristics of the HEAL cohort**

	All^a^	Non-Hispanic	African American	Hispanic
	N(%)	White N(%)	N(%)	N(%)
	**533**	**305**	**151**	**60**
**Study Site**				
Western Washington	99	83	0	2
New Mexico	283	222	0	58
Los Angeles	151	0	151	0
**Body mass index (BMI) (kg/m** ^**2**^ **)**				
Mean (s.d)	27.8 (6.4)	26.5 (5.7)	30.7 (7.4)	27.1 (4.7)
**Age (years)**				
Mean (s.d.)	57.6 (10.7)	60.6 (10.9)	52.4 (7.9)	56.2 (11.3)
**Estrogen receptor (ER) status**				
Negative	109	43	52	13
Positive	375	237	88	36
Unknown	49	25	11	11
**SEER** ^**b**^ **summary stage**				
Local	383	241	85	46
Regional	150	64	66	14
**Treatment at diagnosis**				
Surgery	126	69	37	18
Surgery and radiotherapy	203	138	37	21
Any chemotherapy	204	98	77	21
***GSTM1***				
Null Genotype	280 (52.5)	138 (45.3)	109 (72.2)	28 (46.7)
Positive Genotype	253 (47.5)	167 (54.7)	42 (27.8)	32 (53.3)
χ^2^ = 34.36 P<0.0001				
***GSTT1***				
Null Genotype	424 (79.6)	249 (81.6)	115 (76.2)	49 (81.7)
Positive Genotype	109 (20.4)	56 (18.4)	36 (23.8)	11 (18.3)
χ^2^ = 4.35 P=0.22				
***GSTP1***				
Wildtype (Ile/Ile)	223 (41.8)	137 (44.9)	53 (35.1)	27 (45.0)
Heterozygous (Ile/Val)	250 (46.9)	137 (44.9)	76 (50.3)	27 (45.0)
Homozygous (Val/Val)	60 (11.3)	31 (10.2)	22 (14.6)	6 (10.0)
χ^2^ = 6.16 P=0.41				

^a^ 17 patients were described as ‘other race’; this accounts for the differences in numbers between racial/ethnic subgroups and the overall total.

^b^ Surveillance, Epidemiology and End Results (SEER).

When we compared distribution of genotypes by participants’ characteristics (ER, tumor stage and BMI), carriers of the *GSTM1* null mutation were more likely to have stage 3 tumors (χ^2^=3.87 P=0.05) compared to non-carriers (Table 2). Carriers of the *GSTT1* null mutation were more likely to have ER-negative tumors (χ^2^=4.52; P=0.03). There were no significant associations between the *GSTP1* polymorphism and patient characteristics. When we excluded participants who had surgery only from the analysis, these associations were no longer significant (P=0.06 for both) but this may be due to smaller numbers.

**Table 2:** **Associations between SNPs and participant characteristics**

***All participants N=553***							
	***GSTM1***		***GSTT1***		***GSTP1***		
	Positive	Null	Positive	Null	Ile/Ile	Ile/Val	Val/Val
**BMI (kg/m** ^**2**^ **)**							
<=25	102	116	52	166	95	103	20
	(40.3%)	(41.43%)	(47.7%)	(39.2%)	(42.6%)	(41.2%)	(33.3%)
>25	151	164	57	258	128	147	40
	(59.7%)	(58.6%)	(52.2%)	(60.8%)	(57.4%)	(58.8)	(66.7%)
	χ^2^=0.10 P =0.79		χ^2^=2.62 P =0.11		χ^2^=1.70 P =0.43		
**ER** ^**a**^							
ER-	45	64	15	94	41	57	11
	(19.6%)	(25.2%)	(14.7%)	(24.6%)	(19.8%)	(25.6%)	(20.0%)
ER+	185	190	87	288	166	165	44
	(80.4%)	(74.8%)	(85.3%)	(75.4%)	(80.2%)	(74.3%)	(80.0%)
	χ^2^=2.19 P=0.14		χ^2^=4.52 P=0.03		χ^2^=2.34 P=0.31		
**Stage**							
Local	192	191	75	308	154	188	41
	(75.9%)	(68.2%)	(68.8%)	(72.6%)	(67.1%)	(75.2%)	(68.3%)
Regional	61	89	34	116	69	62	19
	(24.1%)	(31.8%)	(31.2%)	(27.4%)	(30.9%)	(24.8%)	(31.7%)
	χ^2^=3.87 P=0.05		χ^2^=0.63 P=0.42		χ^2^=2.61 P=0.27		
***Omitting patients who received surgery only N=407***							
	***GSTM1***		***GSTT1***		***GSTP1***		
	**Positive**	**Null**	**Positive**	**Null**	**Ile/Ile**	**Ile/Val**	**Val/Val**
**BMI** **(kg/m** ^**2**^ **)**							
<=25	72	89	40	121	72	73	16
	(37.1%)	(41.8%)	(46.5%)	(37.7%)	(40.9%)	(39.5%)	(34.8%)
>25	122	124	46	200	104	112	30
	(62.9%)	(58.2%)	(53.5%)	(62.3%)	(59.1%)	(60.5)	(65.2%)
	χ^2^=0.92 P =0.34		χ^2^=2.21 P =0.14		χ^2^=0.57 P =0.75		
**ER** ^**a**^							
ER-	38	57	13	82	36	50	9
	(20.5%)	(28.6%)	(15.3%)	(27.4%)	(21.4%)	(29.1%)	(20.5%)
ER+	147	142	72	217	132	122	35
	(79.5%)	(71.4%)	(84.7%)	(72.6%)	(78.6%)	(70.9%)	(79.6%)
	χ^2^=3.38 P=0.06		χ^2^=5.23 P=0.02		χ^2^=3.16 P=0.21		
**Stage**							
Local	141	136	59	218	113	134	30
	(72.7%)	(63.9%)	(68.6%)	(67.9%)	(64.2%)	(72.4%)	(65.2%)
Regional	53	77	27	103	63	51	16
	(27.3%)	(36.1%)	(31.4%)	(32.1%)	(35.8%)	(27.68%)	(34.8%)
	χ^2^=3.64 P=0.06		χ^2^=0.01 P=0.91		χ^2^=3.00 P=0.22		

^a^ Omitted 49 participants with unknown ER status’.

Carriers of the *GSTT1* null mutation had significantly higher levels of CRP compared to non-carriers (mean: 4.54 vs. 3.01 mg/L; P=0.01). Levels were also significantly higher in participants with diabetes (mean: 10.23 vs. 2.92 mg/L; P=0.02), but there were no differences in participants without diabetes. In contrast the *GSTM1* null mutation was associated with significantly lower levels of CRP among diabetics only (mean: 4.83 vs. 14.61 mg/dL). There were no associations between *GSTP1* and CRP (data not shown).

No significant association was observed between either *GSTM1* or *GSTT1* null mutations, and breast- or all-cause mortality. Heterozygous carriers of the *GSTP1* Ile^105^Val polymorphism had a significantly increased HR for all-cause mortality, compared to wild-type homozygotes (HR=1.98; 95% CI 1.25-3.12). When examined as a recessive model (all carriers of the variant allele, vs. wild-type homozygotes), the variant allele was associated with an increased risk of all-cause mortality (HR=1.81; 95% CI 1.16-2.82). There was no association between the *GSTP1* Ile^105^Val polymorphism and breast cancer-specific mortality. When we examined risk in women who were postmenopausal at baseline (N=341), HR was similar to that in the entire population for all 3 polymorphisms (data not shown). Finally, we examined associations with mortality in patients who received any treatment (chemotherapy/radiotherapy): there were no differences in associations for *GSTT1* and *GSTM1*. The association for *GSTP1* for homozygous Val/Val changed from HR=1.24 95% CI 0.59-2.58) to HR=0.60 95% CI 0.20-1.75 (Table 3).

**Table 3:** **Associations between the Ile** ^**105**^ **Val polymorphisms in** ***GSTP1*** **, null mutations in** ***GSTM1*** **and** ***GSTT1*** **, and breast-cancer and all-cause mortality**

Genotype	Events/N total	Unadjusted		Full Model^a^		Events/N total	Radiotherapy+Chemotherapy only	
		HR	95% CI	HR	95% CI		HR	95% CI
**Breast cancer Mortality**								
***GSTM1***								
Positive genotype	17/253	1.00	Ref.	1.00	Ref.	14/194	1.00	Ref.
Null genotype	28/280	1.48	0.81-2.71	0.93	0.49-1.79	21/213	0.88	0.41-1.86
**P** ^**b**^			0.21		0.83			0.74
***GSTT1***								
Positive genotype	11/109	1.00	Ref.	1.00	Ref.	8/86	1.00	Ref.
Null genotype	34/424	0.80	0.40-1.60	0.86	0.43-1.72	27/321	0.91	0.40-2.03
**P** ^**b**^			0.54		0.68			0.80
***GSTP1***								
Ile/Ile	16/233	1.00	Ref.	1.00	Ref.	14/176	1.00	Ref.
Ile/Val	26/250	1.53	0.82-2.86	1.66	0.87-3.16	19/185	1.37	0.67-2.84
Val/Val	3/60	0.75	0.22-2.58	0.63	0.17-2.27	2/46	0.42	0.10-2.12
**P** ^**b**^			0.71		0.82			0.74
***GSTP1***								
Ile/Ile	16/223	1.00	Ref.	1.00	Ref.	14/176	1.00	Ref.
Val/Val + Val/Ile	29/310	1.38	0.74-2.55	1.43	0.76-2.68	21/321	1.17	0.58-2.37
**P** ^**b**^			0.30		0.27			0.67
**All cause Mortality**								
***GSTM1***								
Positive genotype	45/208	1.00	Ref.	1.00	Ref.	31/194	1.00	Ref.
Null genotype	54/226	1.07	0.71-1.58	0.88	0.57-1.34	38/213	0.77	0.45-1.30
**P** ^**b**^			0.76		0.55			0.32
***GSTT1***								
Positive genotype	17/109	1.00	Ref.	1.00	Ref.	12/86	1.00	Ref.
Null genotype	82/424	1.21	0.71-2.03	1.22	0.72-2.08	57/321	1.33	0.71-2.51
**P** ^**b**^			0.48		0.47			0.38
***GSTP1***								
Ile/Ile	30/223	1.00	Ref.	1.00	Ref.	24/176	1.00	Ref.
Ile/Val	59/250	**1.89**	**1.21-2.95**	**1.98**	**1.25-3.12**	39/185	**1.72**	**1.01-2.93**
Val/Val	10/60	1.41	0.68-2.89	1.24	0.59-2.58	6/46	0.60	0.20-1.75
**P** ^**b**^			0.05		0.08			0.72
***GSTP1***								
Ile/Ile	30/223	1.00	Ref.	1.00	Ref.	24/176	1.00	Ref.
Ile/Val + Val/Val	69/310	**1.80**	**1.16-2.77**	**1.81**	**1.16-2.82**	45/231	1.47	0.87-2.47
**P** ^**b**^			0.008		0.008			0.15

^a^ Adjusted for race/ethnicity/study-site; BMI (categorical <18.5 kg/m^2^; ≥18.5 and <25 kg/m^2^; ≥25 and <40 kg/m^2^; ≥40 kg/m^2^); SEER summary tumor stage (local vs. regional) and treatment received at diagnosis (surgery; surgery+radiotherapy; chemotherapy).

^b^ Wald test for trend.

Boldface type indicates a statistically significant result.

We performed a 3-way gene analysis, examining combinations of *GSTM1* null/present; *GSTT1* null/present and *GSTP1*^105^Ile/Ile vs. Ile/Val+Val/Val. An increased risk of all-cause mortality was observed for each group relative to the referent (*GSTM1* present/*GSTT1* present/*GSTP1*^105^Ile/Ile), but none reached statistical significance (data not shown). Breast cancer-specific mortality demonstrated a different pattern, with carriers of *GSTT1* null mutation/*GSTM1* present/*GSTP1*^105^Ile/Ile genotypes associated with a reduced risk (HR=0.12; 95% CI 0.01-1.16) compared to participants with *GSTM1* present/*GSTT1* present/homozygous wild-type for *GSTP1*. However this association was not significant (P=0.06).

We next analyzed the same endpoints for *GSTP1*, *GSTM1* and *GSTT1* polymorphisms in patient subgroups, using fully adjusted models. The *GSTP1* Val allele was associated with an approximate 2-fold increased risk of all-cause mortality in patients with ER-positive tumors, compared to those with ER-negative, though this was not significant (P=0.08). There was no evidence of effect modification for other subgroups examined. Due to limited power we did not examine associations for breast cancer-specific mortality in these subgroups.

## Discussion

There are few studies on the role of GST isoenzymes on mortality in breast-cancer survivors drawn from community practice. As described earlier, the majority of these studies had small sample sizes, were based on participants diagnosed prior to 1999, and on women undergoing chemotherapy and/or radiotherapy. In addition, most examined only one GST gene (usually *GSTP1*).

Here we report that the variant Val allele of *GSTP1* (rs1695) was associated with increased risk of all-cause mortality, but not breast cancer-specific mortality, in a cohort of 533 breast-cancer survivors. The amino acid substitution in *GSTP1* Ile^105^Val, results in an enzyme with altered activity, (Ali-Osman et al. 1997) including towards alkylating anticancer agents, (Hayes & Strange 2000; Pandya et al. 2000; Srivastava et al. 1999) and the decreased risk of mortality in carriers of the variant allele who receive chemotherapy may be attributable to longer exposure to the active agent in therapy. Patients homozygous for the variant allele had a lower risk of chemo-resistance when treated with doxorubicin (OR= 0.11; 95% CI 0.01-0.90; P=0.04) (Romero et al. 2012). However, when we examined the association of *GSTP1* polymorphisms in subgroups of patients, we found no association between the polymorphism and treatment received.

In contrast, deletions in the *GSTM1* or *GSTT1* genes were not associated with mortality confirming results from one other study, (Yang et al. 2005) but not in another, (Ambrosone et al. 2001) though the latter studied women recruited between mid 1980s-1990s.

We also found an association between breast cancer-specific mortality with carriers of *GSTT1* null mutation/*GSTM1* present/*GSTP1* Ile/Ile genotypes associated with a reduced risk of breast cancer-specific mortality compared to participants with *GSTM1* present/*GSTT1* present/homozygous wild-type for *GSTP1*. While this was not significant, we cannot discount inadequate power.

When we examined associations in patients who received any treatment (chemotherapy/radiotherapy; excluding those who received surgery only), homozygous carriers of the *GSTP1* Val allele had a decreased risk of all-cause mortality, although this was not significant, compared to all participants. This is similar to another report in Chinese women who all received adjuvant chemotherapy; (Yang et al. 2005) another reported that the *GSTP1* Val/Val polymorphism was non-significantly associated with worse overall survival in women treated with high-dose chemotherapy (Bewick et al. 2008).

We found significantly lower levels of CRP in carriers of the *GSTM1* null mutation in patients with diabetes only; and higher levels of CRP in carriers of the *GSTT1* null mutation; the latter differed from results in patients with diabetes and smokers (lower CRP among carriers of the *GSTT1* null mutation); (Hayek et al. 2006; Miller et al. 2003) however these participants were otherwise healthy.

Limitations of our study include relatively small numbers of events, thus we were not able to evaluate the association with outcome by subgroups such as menopausal status; and three-way gene analyses are underpowered. We also had limited power to examine breast cancer-specific mortality. The cohort was established before some current treatments such as aromatase inhibitors and Her2/*neu* targeted therapies were available, and therefore we cannot estimate what associations GST isoenzymes might have with survival in women using these treatments. There was also a possible selection bias in this study. As blood for genotyping was obtained approximately 30-months post-diagnosis, and we excluded participants who were under treatment for recurrence, associations with early breast-cancer mortality would not be observed, and it is possible that genotype may have stronger associations closer to the time of diagnosis.

However, our study has a larger sample size than most prior studies examining the association between GST polymorphisms and survival, and participants also underwent more contemporary treatment protocols. Given the heterogeneity of published studies (different therapies, stages of disease, and recruitment periods), suggestions for further study include examining larger studies of pooled data of women diagnosed at similar time-periods who underwent similar treatment regimens, thus enhancing power to detect associations between GST isoenzymes and longer-term survival.
